# Computational Identification of Novel Transcriptional Regulators and Functional Gene Clusters in *Lactococcus lactis* Using Integrated Bioinformatics Approaches

**DOI:** 10.3390/microorganisms14071594

**Published:** 2026-07-22

**Authors:** Ekaterina Wolf, Tatiana Sokolova, Ilya Akberdin, Aleksey Sazonov

**Affiliations:** Scientific Center of Genetics and Life Sciences, Sirius University of Science and Technology, Sirius 354340, Russia; volf.katya.0308@gmail.com (E.W.); akberdin.ir@talantiuspeh.ru (I.A.);

**Keywords:** *Lactococcus lactis*, gene regulatory networks, systems microbiology, stress response, WxL operon

## Abstract

*Lactococcus lactis* is an essential industrial “cell factory” used extensively in food fermentation and biotechnology. However, a critical biological question regarding the regulatory mechanisms of the microorganism’s adaptation process remains unresolved: how does the bacterium transcriptionally coordinate the trade-off between primary metabolism and cell-surface remodeling during environmental stress and competence? To date, a unified, global model of its gene regulatory networks (GRNs) that accounts for this transition remains lacking. To address this fragmentation and eliminate selection bias, we integrated the complete compendium of publicly available transcriptomic datasets for *L. lactis* deposited in the NCBI database as of the summer of 2025. This exhaustive dataset encompasses a wide range of conditions, including thermal, acid, and phage-induced stress, as well as natural competence, providing the necessary transcriptional variance for robust network inference. We implemented an integrated bioinformatics pipeline using the GENIE3 algorithm to infer a core regulatory network common to all tested conditions, complemented by an ensemble of DeepTFactor, Entraf, and p2TF tools to predict strain-specific potential transcription factors (TFs) for *L. lactis*. The co-expression network partitioned into 50 functional clusters, notably highlighting putative regulators for a unique WxL operon potentially involved in cell-surface modifications. Furthermore, we proposed candidate regulatory targets for the master competence regulator, ComX, and computationally predicted CpsY as a potential LysR-family regulator of branched-chain amino acid metabolism. These findings provide a transcriptomics-based computational model of *L. lactis* regulation. By clearly distinguishing between established regulatory pathways and purely computational predictions, we suggest several uncharacterized proteins as putative key nodes in the bacterial response to environmental challenges. While requiring direct experimental validation to establish physical interactions, this computational approach generates high-confidence hypotheses and offers a curated resource of candidates for targeted metabolic engineering.

## 1. Introduction

*Lactococcus lactis* subsp. *lactis* is a Gram-positive bacterium of paramount importance in the dairy industry, where it serves as a primary starter culture for cheese and fermented milk production [1,2,3]. Beyond its traditional role, it is increasingly utilized as a “cell factory” for producing recombinant proteins, vitamins, and therapeutic molecules due to its generally recognized as safe (GRAS) status [4] and the absence of endotoxins [5]. However, the efficiency of these industrial processes is often compromised by environmental stressors, such as rapid acidification, temperature fluctuations, and osmotic pressure, which limit bacterial growth and metabolic activity [6,7,8]. Survival in these fluctuating environments requires a swift and coordinated genetic response. While comparative genomic approaches have outlined core conserved regulons in lactic acid bacteria [9] and some global regulators, such as CodY and CcpA, have been well-characterized for their roles in nitrogen metabolism and carbon catabolite repression during growth in milk [10,11,12,13], many components of the *L. lactis* regulatory landscape remain “dark nodes” or poorly annotated. For example, in a recent large-scale predictive study utilizing the ESM2 protein language model, only four master global regulators (CcpA, Spx, Nnr, and CmbR) were predicted for *Lactococcus lactis* [14]. Notably, only CcpA and Spx are currently present in the RefSeq annotation [14]. While this constraint highlights the stringency of model-driven global regulator discovery compared to standard model organisms like *E. coli* (for which 32 global TFs were predicted), it stands in sharp contrast to the approximately 181 total site-specific transcriptional regulators currently annotated in the RefSeq database for *L. lactis*. This massive gap underscores the necessity of dynamic, expression-based network inference to uncover the functional roles of the remaining uncharacterized regulatory nodes. Traditional experimental methods for identifying transcription factors and their targets are resource-intensive, leading to a significant gap between the available transcriptomic data and our understanding of the underlying regulatory logic. Advances in systems microbiology and machine learning now allow for the in silico reconstruction of Gene Regulatory Networks (GRNs) by analyzing co-expression patterns across diverse experimental conditions [15,16,17,18]. Techniques such as tree-based ensemble methods (e.g., GENIE3) have proven effective in identifying non-linear interactions between regulators and their target genes [19]. The specific regulatory problem addressed in this study is the identification of core regulatory hubs that govern cell-wall permeability and stress adaptation, which cannot be isolated from single-condition experiments. To successfully infer a robust co-expression network, it is essential to capture the maximum feasible phenotypic and transcriptional variance. Therefore, we purposefully retrieved and integrated a compendium of 38 *L. lactis* subsp. *lactis* transcriptomic datasets available in the NCBI database up to the end of 2025. These specific public datasets—encompassing orthogonal perturbations such as thermal, acid, and phage-induced stresses, as well as natural competence—were selected because they force the GENIE3 algorithm to identify universal regulatory modules rather than isolated, stress-specific artifacts. This environmental heterogeneity provides the necessary statistical power to distinguish robust transcriptome-supported inferences from condition-specific transcriptional noise.

Rather than claiming to present a definitive physical interactome, our goal is to establish a data-driven predictive framework that distinguishes established biological knowledge from novel computational hypotheses. We acknowledge that definitive support for these novel regulatory interactions will ultimately require specific biochemical evidence, such as protein–DNA binding assays. Until such validation is performed, our model serves to generate high-confidence regulatory hypotheses. By consolidating this global transcriptomic data, we computationally inferred a network comprising 1791 genes and 98 potential transcription factors. Our analysis specifically focused on identifying putative novel functional modules, such as the WxL operon involved in surface protein anchoring [20], and computationally predicting the regulatory shifts occurring during competence induction and stress response. These results provide a transcriptome-supported perspective on the adaptive potential of *L. lactis* and offer specific candidate targets for follow-up experimental validation and strain optimization.

## 2. Materials and Methods

### 2.1. Data Sources and Experimental Conditions

A total of 38 transcriptomic datasets for *Lactococcus lactis* subsp. *lactis* were integrated into this study to reconstruct the gene regulatory network (GRN). These data were obtained from public repositories, including the NCBI BioProject database, and covered four major experimental categories:

• Acid stress: Analysis of strains F44 and G423 under pH 4.0 and control pH 7.0 conditions [21]. These strains typically express the arginine deiminase pathway, providing an inherent degree of acid tolerance.
• Thermal stress: Data from commercial L. lactis biovar diacetylactis strain SD96, along with its thermotolerant derivatives (RD01 and RD07) generated through adaptive laboratory evolution (ALE) by prolonged growth at high temperatures in UHT milk [22].
• Phage infection: Transcriptomic profiles 2 and 15 min post infection [23].
• Genetic perturbations: Experiments involving the deletion of global regulators codY and covRS, as well as the overexpression of the competence regulator, comX [10].

By deliberately integrating these distinct genetic backgrounds and their derivatives (e.g., combining subsp. lactis with biovar diacetylactis backgrounds), we maximized the transcriptional and phenotypic variance within the input compendium.

To mitigate potential batch effects and ensure high comparability between data originating from different studies and platforms, all integrated datasets were re-processed from raw reads using a strictly standardized computational protocol, as detailed in Section 2.3 and Section 2.4. A comprehensive metadata manifest detailing the study titles, exact GEO/SRA accession numbers, specific condition labels, and the overall read alignment rates for each of the 38 individual datasets is provided in Supplementary Table S2.

### 2.2. Identification of Potential Transcription Factors

**Figure 1:**
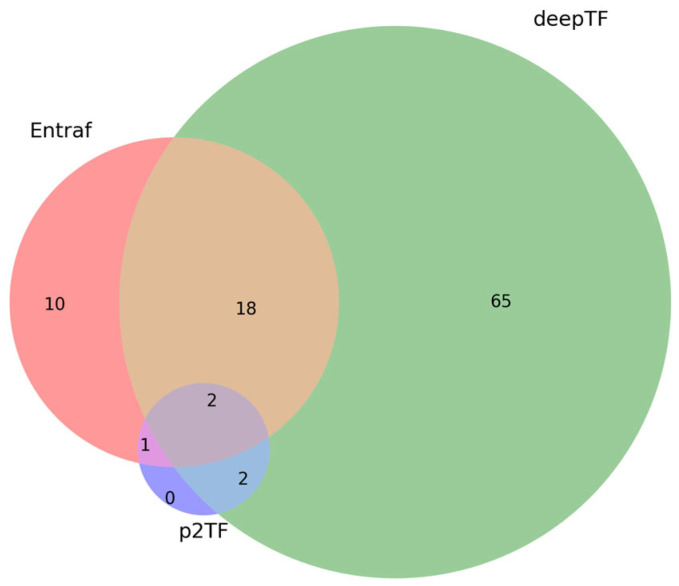
Overlapping of 98 potential TFs for *L. lactis* subsp. *lactis* from three databases: DeepTFactor, Entraf, and P2TF.

Complementary to the data collection, potential TFs were identified using an ensemble computational strategy. This approach utilized deep learning-based tool DeepTFactor (v.1.0) [24], motif-oriented algorithm Entraf (accessed 15 September 2025, https://entraf.iimas.unam.mx/index.php) [25], and domain-search method p2TF (accessed 15 June 2024, http://www.p2tf.org/) [26] (Figure 1). A final non-redundant list of 98 potential TFs was compiled by merging results from these tools, ensuring maximum coverage of the organism’s regulatory landscape and included in the subsequent analyses.

### 2.3. RNA-Seq Processing and Differential Expression Analysis

Raw sequencing reads were processed through a standardized bioinformatics pipeline to ensure consistency across datasets. Initial quality assessment was performed using FastQC (v0.12.0) [27], followed by adapter trimming and the removal of low-quality bases with fastP (v0.23.4) [28]. The resulting high-quality reads were aligned to the reference genome of *Lactococcus lactis* subsp. *lactis* (ASM317683v1) using Bowtie2 (v2.5.4) [29]. Gene-level expression quantification was subsequently conducted using featureCounts (v2.0.6) based on the RefSeq annotation (GCF_003176835.1). Statistical analysis of differential expression was carried out in the R environment (v4.4.1) using the DESeq2 package (v1.46.0) [30]. Genes were classified as differentially expressed genes (DEGs) based on a threshold of |log_2_FoldChange| > 1 and a Benjamini–Hochberg adjusted *p*-value of less than 0.05. The correlation between samples was evaluated to assess the underlying data structure (Figure S1). As expected for an integrated compendium, the initial clustering pattern was primarily driven by the study of origin. Rather than viewing this strictly as a technical artifact, this clustering reflects the distinct, condition-specific biological responses (e.g., pH stress vs. thermal stress) captured by each study. Consequently, this observation directly informed our downstream network strategy: aggressive batch-effect correction was deliberately avoided to prevent the erasure of crucial cross-condition biological variance, justifying the use of median-of-ratios normalization to handle sequencing-depth differences while retaining the native expression variance required for random forest inference.

### 2.4. Network Inference and Modular Analysis

Expression matrix construction began with the filtering of low-expressed genes; only genes with a minimum of 10 mapped reads in samples were retained to reduce technical noise. The raw count matrix was then normalized using DESeq2’s median-of-ratios method. Specifically, size factors were estimated utilizing an intercept-only design (~1), which robustly accounts for varying sequencing depths and compositional biases across the heterogeneous datasets without imposing condition-specific models. Explicit batch-effect correction was intentionally omitted to preserve the cross-condition biological variance, which the GENIE3 random forest algorithm (being mathematically scale-invariant and driven by relative expression ranking) inherently leverages to establish robust regulatory links common across the tested conditions. For GENIE3 (v1.28.0) execution, the random seed was fixed (set.seed(123)) to ensure algorithmic reproducibility, and the ensemble was built using 1000 trees. The raw GENIE3 weight matrix was sparsified to retain the top 4000 regulatory edges. To justify this threshold and ensure the robustness of our biological conclusions, a topological sensitivity analysis was performed at adjacent edge cutoffs (top 3000 and 5000 edges) and varying clustering resolutions. The 4000-edge cutoff heuristically provided the optimal balance: lower thresholds (3000) led to excessive network fragmentation and premature exclusion of peripheral genes in the WxL and competence modules, while higher thresholds (5000) caused module blending due to edge inflation and false-positive background noise. Most importantly, the core structure of our primary functional modules (e.g., the CpsY–CodY–Ilv axis and the WxL operon) remained topologically stable across all tested cutoffs. Hierarchical clustering of the active network was performed using the Euclidean distance derived from the GENIE3 topological weights, applying Ward’s minimum variance method (ward.D2). Ward’s method was specifically chosen because it minimizes intra-cluster variance, thereby yielding compact, densely connected regulatory modules. The resulting dendrogram was cut into 50 distinct clusters. To ensure the results were not overly sensitive to this parameter, we qualitatively assessed alternative clustering resolutions (e.g., k = 30 and k = 65). A lower resolution (k = 30) forced distinct functional pathways (e.g., amino acid biosynthesis and carbohydrate metabolism) to merge non-specifically, whereas a higher resolution (k = 65) began to artificially fragment well-established operons into separate sub-clusters. Thus, k = 50 provided the optimal, biologically coherent partitioning resolution. This clustering sensitivity analysis, combined with the 4000-edge topological threshold evaluation, ensures that the identified functional modules are robust to parameter variations. Functional annotation of these clusters was performed by integrating data from the Clusters of Orthologous Groups (COG; 2020 Update, accessed 12 August 2025), Kyoto Encyclopedia of Genes and Genomes (KEGG; Release 115.0, accessed 10 August 2025), and Gene Ontology (GO; Release 2025-08, accessed 10 August 2025) databases, alongside detailed domain analysis using RefSeq and PFAM (v37.0, accessed 12 August 2025). While the GENIE3 algorithm robustly infers the topological presence and importance of regulatory interactions, it does not natively predict the mode of regulation (activation versus repression). To computationally estimate regulatory directionality and contextualize the network, we overlaid condition-specific differential expression data (log_2_FoldChange) onto the inferred network topology. By analyzing the concurrent expression shifts (up- or down-regulation) of connected nodes—such as a predicted regulator and its targets—under specific environmental perturbations, we qualitatively inferred whether an edge likely represents putative activation or repression within that specific biological context. We acknowledge, however, that these directional assignments are predictive; steady-state transcriptomic data may obscure complex regulatory dynamics, meaning true mechanistic directionality ultimately requires independent experimental validation. Structural visualization and topological analysis of the reconstructed network were performed following our previously validated pipeline [31], utilizing the Cytoscape software environment (v3.10.2) [32].

### 2.5. Operon Prediction and Regulatory Motif Discovery

The structural organization of the genome within the network was refined using the Operon-mapper Web server (accessed 20 December 2025, https://biocomputo.ibt.unam.mx/operon_mapper/) [33]. For *cis*-regulatory element and motif analysis, promoter windows were strictly defined as the sequence regions ranging from −300 to +50 bp relative to the annotated translation start site. Motif discovery via the XSTREME Web server (v5.5.5, accessed 13 February 2026, https://meme-suite.org/meme/tools/xstreme) [34] utilized a background model generated by shuffling the input sequences (0-order Markov model), which preserves local sequence composition while disrupting true functional motifs. The statistical significance of de novo discovered motifs was strictly evaluated using an E-value threshold of <0.05, as detailed in the raw computational outputs provided in Supplementary Tables S6–S8. To control the false discovery rate (FDR) and biologically validate these computational predictions, the discovered motifs were subsequently aligned against the “Combined Prokaryotes” known motif database using Tomtom (Amsterdam, The Netherlands). Only motif alignments maintaining an FDR-adjusted q-value < 0.05 were considered robust and retained for downstream network annotation.

To ensure full computational reproducibility, a comprehensive record of all software versions, exact analytical parameters, database releases, and Web-server access dates used in this study is provided in Supplementary Table S9.

## 3. Results

### 3.1. Global Topology and Reconstruction of the L. lactis Co-Expression Network

We applied the GENIE3 algorithm with T = 1000 trees for the collected datasets. The K parameter (the number of candidate regulators randomly selected at each split) was set to the square root of the total number of input regulators (K = √p), following the default random forest setting. The resulting GENIE3 co-expression network includes the top 4000 interactions, representing approximately 70% of the genome of *Lactococcus lactis* subsp. *lactis* (1791 genes). Among these genes, 98 are transcriptional regulators robustly predicted by our ensemble approach (p2TF, DeepTFactor, and Entraf) (Table S1). While the RefSeq annotation for *L. lactis* subsp. *lactis* identifies approximately 181 total regulators, the GENIE3 algorithm strictly relies on cross-condition expression variance to establish topological links. Consequently, regulators that were unexpressed or statically expressed or lacked sufficient transcriptional variance across the 38 integrated conditions could not mathematically form confident edges and were naturally excluded. Thus, the 98 retained TFs represent the actively functioning regulatory core under the studied environmental stresses. Table S2 summarizes information on the samples, including their sources and accession IDs.

**Figure 2:**
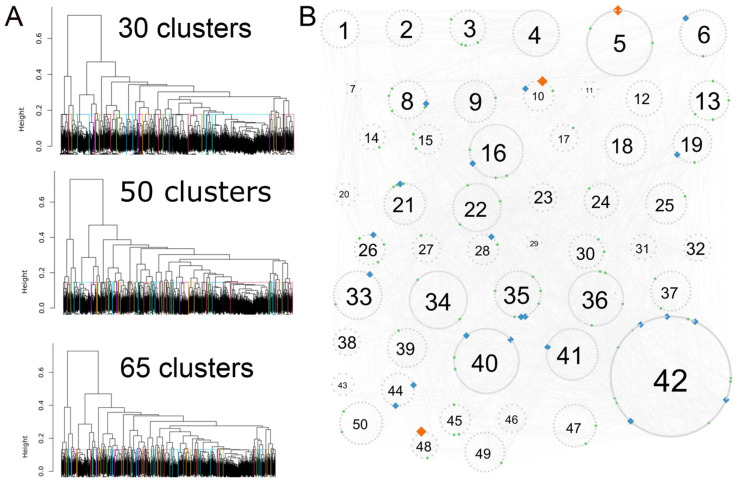
Summary of co-expression network structure. (**A**) Three dendrograms show the results of the Ward.D2 algorithm, dividing genes into 30, 50, and 65 clusters. Colored blocks unite the genes within each cluster. (**B**) Circles represent clusters constructed using the Ward.D2 algorithm. The radius of each circle reflects the number of genes it contains. Diamonds indicate putative TFs predicted by one (green), two (blue), or three (orange) tools. Small circles correspond to individual genes. The network comprises 1791 genes and 98 putative TFs, organized into 50 functional clusters.

The global co-expression structure was partitioned into 50 functional modules using the Ward.D2 hierarchical clustering algorithm (Figure 2A,B). The biological validity of this partitioning is underscored by the high functional homogeneity of the resulting clusters. For instance, Cluster 18 exhibits near-perfect enrichment for ribosomal proteins and translation machinery, serving as an internal “gold standard” that confirms the network’s ability to capture fundamental physiological co-regulation.

### 3.2. Modular Architecture and Functional Landscapes

**Figure 3:**
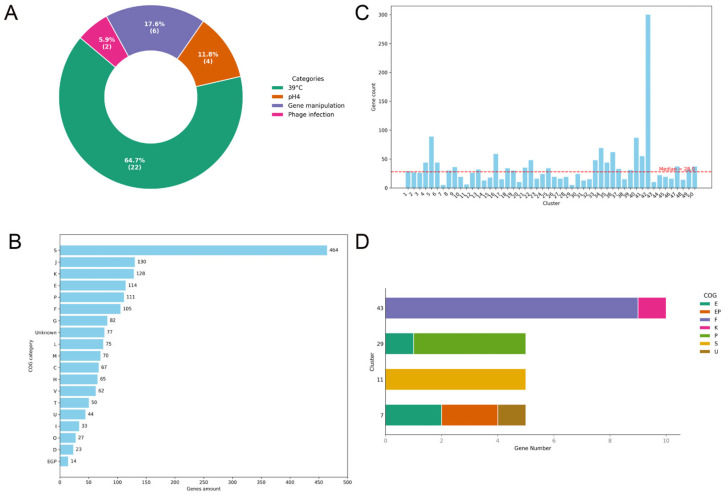
(**A**) The number of samples in each condition: pH4, heat shock (39 °C), gene manipulation (*comX* overexpression, Δ*codY*, and Δ*covRS*), and bacteriophage infection. (**B**) Gene amount in each COG category represented in the network. Categories with fewer than 14 genes are filtered. The “Unknown” category represents genes with no assigned COG annotation. (**C**) Amount of genes in each cluster in the network; the red line shows the median value. (**D**) Clusters containing one, two, or three COG categories.

The co-expression network includes 38 samples and 4 different conditions (pH4, heat shock (39 °C), gene manipulation (*comX* overexpression, Δ*codY*, and Δ*covRS*), and bacteriophage infection) (Figure 3A). The reconstructed network exhibits a highly modular architecture, with cluster sizes following a power-law-like distribution (median size: 28 genes), reflecting a balance between broad metabolic hubs and specialized regulatory units (Figure 3C). The genes in the co-expression network were annotated for further analysis using the COG, KEGG, and RefSeq databases (Table S3). The majority of genes belong to category S (unknown function), followed by J (translation, ribosomal structure, and biogenesis), K (transcription), and E (amino acid transport and metabolism), indicating substantial changes in regulator activity and overall cell metabolism (Figure 3B). Cluster 11 is exclusively enriched for COG category S (unknown function). Two clusters contain two different COG categories (Figure 3D): cluster 29 includes categories related to inorganic ion transport and metabolism, as well as amino acid transport and metabolism. Additionally, cluster 29 is entirely uniform in terms of the metabolic pathways involving its genes (map02010—ABC transporters). In cluster 43, 9 out of 10 genes correspond to nucleotide transport and metabolism, and the remaining gene belongs to category K (transcription) (Figure 3D).

Overall, many of the observed changes are associated with the metabolism and transport of various biological molecules. Additionally, alterations in transcription point to the important role of transcriptional regulators in this analysis (Table S4).

To validate the biological relevance of this modular structure, we benchmarked the network against three experimental transcriptomic datasets representing critical nodes in the *L. lactis* regulatory hierarchy: CodY repressor knockout (Δ*codY*); CovRS (Δ*covRS*) stress-response system deletion; and the overexpression of the competence master regulator, *comX* (*comX* overexpression). These three genes are interconnected, as *codY* and *covRS* are repressors of the *comX* regulator [9] (Figure S2). These datasets provide a rigorous framework to evaluate how the inferred modules respond to major genetic perturbations in the CodY/CovRS/ComX axis, which governs the transition between growth, stress adaptation, and potential competence development [10].

### 3.3. Identification and Characterization of the WxL Operon (Cluster 11)

One of the most intriguing findings in the reconstructed network is with respect to Cluster 11, which comprises six genes (LL14B4_RS08305, LL14B4_RS08295, LL14B4_RS08310, LL14B4_RS08300, LL14B4_RS13575, and LL14B4_RS13315). These genes are not functionally annotated in RefSeq. However, the available descriptions indicate that four of them (LL14B4_RS08305, LL14B4_RS08295, LL14B4_RS08310, and LL14B4_RS08300) encode proteins containing WxL-domain surface cell-wall-binding motifs. Furthermore, reconstruction of the operon structure using OperonMapper revealed that these WxL genes likely form a single operon. The remaining two genes in the cluster lack any functional annotation. WxL proteins are of particular interest because their role in competence-related processes remains poorly characterized, though they may have an influence in the context of competence mechanisms and with respect to many cellular functions, such as cell-wall permeability or biofilm formation [20,35,36].

**Figure 4:**
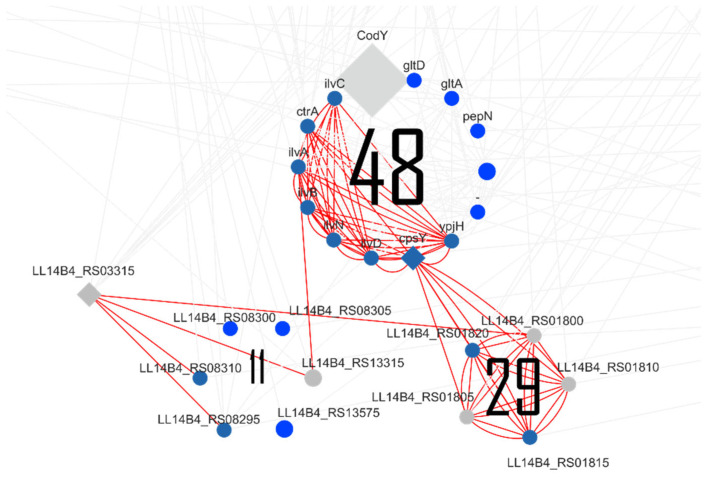
Relations between WxL genes (cluster 11), a potential TF (LL14B4_RS03315), uncharacterized genes of Cluster 29, the CpsY TF, and the *ilv* operon form cluster 48. Red lines indicate links between the selected nodes and each node represents a gene. The blue color of corresponding nodes signifies downregulation of a certain gene under the *comX*-overexpressing condition.

Topologically, Cluster 11 exhibits a unique “coordinated satellite” behavior: while direct edges between the cluster members are sparse in the top-weighted 4000 interactions, they share a highly congruent neighborhood, being collectively linked to a specific regulatory hub. Notably, one transcription factor, LL14B4_RS03315, is associated with three genes from cluster 11 (Figure 4). According to the RefSeq annotation, this protein belongs to the XRE family of transcriptional regulators. It is also connected to another putative transcription factor, LL14B4_RS08280, which is likewise annotated as an XRE-family protein. In addition, cluster 11 is connected to genes encoding two peptidases (LL14B4_RS09535, *pepO*; LL14B4_RS11510, *pepX*); a mucin-binding protein (LL14B4_RS03310); *dapB* (LL14B4_RS08190), which encodes 4-hydroxy-tetrahydrodipicolinate reductase essential for lysine biosynthesis; and *ilvA* (LL14B4_RS06025), encoding threonine dehydratase involved in L-isoleucine biosynthesis. Three additional connected genes (LL14B4_RS00840, LL14B4_RS08710, and LL14B4_RS10705) remain uncharacterized.

Overall, genes associated with Cluster 11 are enriched in KEGG pathways such as map01230 (amino acid biosynthesis) and map01110 (biosynthesis of secondary metabolites), suggesting potential regulatory links between membrane-associated WxL proteins and cellular biosynthetic processes. Analysis of gene expression in the *comX* overexpression strain revealed that only the WxL genes, along with *dapB* and *ilvA*, are downregulated, whereas the expression of other genes—including putative transcription factor LL14B4_RS03315—remains unchanged (Table S5). In contrast, in the ∆*covRS* strain, one of the putative transcription factors, LL14B4_RS08280, is upregulated (log_2_FoldChange = 1.98).

**Figure 5:**
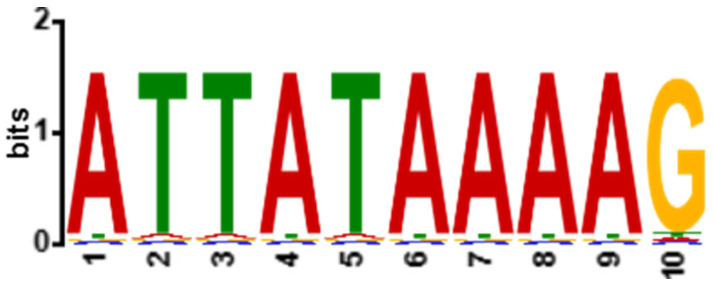
The common binding motif of WxL genes.

It is worth noting that according to the regulatory motif discovery, all WxL genes share a common binding motif, i.e., ATTATAAAAG (Figure 5), which closely resembles the consensus binding sequences described for PerR-like regulators in *B. subtilis*, MogR in *L. monocytogenes*, and Zur in *P. protegens*. The other detected motifs are presented in Table S6. The most interesting homolog is PerR in *B. subtilis*, as it regulates genes involved in the inducible peroxide stress response [37], which may also play a role in competence regulation.

This novel cell-surface protein may represent a key component of an as-yet unknown mechanism activated during the competence state. In *L. lactis*, the thick peptidoglycan layer acts as a physical barrier to DNA uptake during competence. The observed downregulation of cell-wall-binding WxL proteins and amino acid biosynthetic genes (involved in peptidoglycan precursors) during *comX* overexpression suggests a targeted peptidoglycan remodeling mechanism. While highly speculative and requiring direct experimental verification via gene knockout and/or overexpression, we hypothesize that the temporary reduction in WxL-mediated surface anchoring may lead to a loosening or localized weakening of the cell wall, potentially facilitating the assembly of the DNA transformation machinery.

### 3.4. Expansion of the ComX Regulon and Natural Competence

To characterize the systemic impact of competence induction, we analyzed the transcriptional landscape of the *comX* overexpression condition. The perturbed state enabled the identification of additional significant changes in gene activity, including novel potential regulators that may interact with the ComX circuitry either directly or indirectly, as well as potential target genes of previously characterized global regulators.

**Figure 6:**
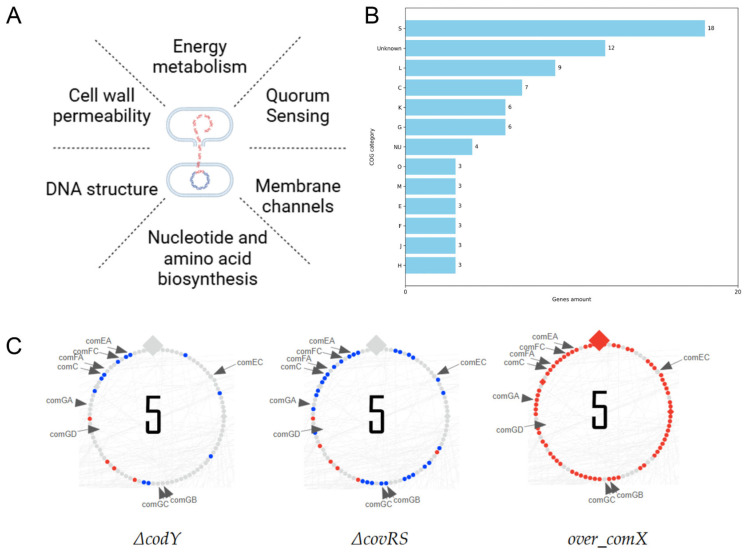
(**A**) Functional categories affected by competence activation in the bacterium. (**B**) Distribution of COG functional categories within Cluster 5. The plot shows the number of genes assigned to each category; categories represented by fewer than three genes are excluded. The “Unknown” category represents genes with no assigned COG annotation. (**C**) Cluster 5 under Δ*codY*, Δ*covRS*, and *comX* overexpression conditions. Red nodes indicate upregulated genes, while blue nodes designate downregulated genes in a certain condition. Circles represent genes, and squares denote potential TFs. Arrows mark *com* genes within the cluster.

ComX is a global regulator of competence and other processes associated with the competent state, including amino acid and nucleotide metabolism, quorum sensing, cell-wall permeability, energy metabolism, and DNA homeostasis (Figure 6A) [38]. Our co-expression network captures gene expression changes across all of these functional transitions. Cluster 5, involving 89 genes, is particularly notable, as more than 90% of its genes exhibit increased activity (log_2_FoldChange > 1). This cluster contains nearly all *com* genes involved in competence, including LL14B4_RS11980 (*comX*, regulator), LL14B4_RS11845 (*comGD*), LL14B4_RS11850 (*comGC*), LL14B4_RS11855 (*comGB*), LL14B4_RS11860 (*comGA*), LL14B4_RS09305 (*comEC*), LL14B4_RS13695 (*comFC*), LL14B4_RS05360 (*comFA*), LL14B4_RS09310 (*comEA*), and LL14B4_RS11400 (*comC*). The membrane complex of Com proteins is shown in Figure S3.

Additionally, the cluster includes 23 genes of unknown function, three genes from the *rec* operon involved in DNA stabilization, and the entire *atp* operon (seven genes). The co-activation of ATP synthase genes and the competence system indicates a preparation of energy metabolism to provide for the high ATP requirements of DNA uptake processes.

In the *covRS* deletion, the activity of Cluster 5 genes is predominantly decreased (log_2_FoldChange < –1), while under *codY* deletion, expression levels remain largely unchanged (Figure 6C). This indicates that the CovRS system is the primary regulator of this module under the studied conditions. The analysis of binding motifs among the genes from Cluster 5 revealed three binding motifs common to 32, 31, and 21 sequences, respectively (Table S7). One of these motifs matches the CodY transcription factor (ATTCATAAA), underscoring its potential role in competence processes.

The broad functional diversity of genes within Cluster 5, together with the presence of *com* operons, highlights the extensive and complex interplay between competence and other cellular processes (Figure 6B).

The co-expression network reveals a marked decrease in the activity of several biosynthetic pathways upon *comX* activation, indicating a redistribution of cellular metabolic resources. Cluster 31 contains 13 genes, 9 of which belong to histidine metabolism [39] and are downregulated (log_2_FoldChange < −2.4). Similarly, Cluster 38, involved in sulfur-containing amino acid metabolism and featuring NlpA-family lipoproteins, shows a similar downward trend. The most dramatic repression was observed in Cluster 48, where the *ilv* genes (BCAA biosynthesis) exhibit an average log_2_FoldChange < −5. These genes are topologically linked to CodY and the CpsY transcription factor, the latter of which is also repressed under *comX* overexpression. Given that BCAA deficiency is a known signal for CodY-mediated derepression, this CpsY–CodY–Ilv axis likely constitutes a regulatory feedback mechanism during competence.

Overall, primary metabolism is repressed in the cell during the competence state in order to conserve resources for survival under stress conditions, although some genes are upregulated. Analyzing the E (amino acid transport and metabolism) and F (nucleotide transport and metabolism) COG categories, the potential transcription factor (LL14B4_RS10805) is described in RefSeq as a positive regulator of the macromolecule biosynthetic process and is connected to several unknown genes (LL14B4_RS08725, LL14B4_RS12775, LL14B4_RS03680, and LL14B4_RS08455) and two periplasmic proteins (YhcA LL14B4_RS03485 and GpsA LL14B4_RS07200). In category F, two genes—*nucA* (LL14B4_RS05380) and *nrdF* (LL14B4_RS05085)—have a log_2_FoldChange greater than 1; both, according to the RefSeq description, are involved in the catabolic nucleotide process. Another activated biosynthesis pathway is the production of ATP. These genes (*atpABCDEF* and *atpH*) are located in the same cluster as the *com* genes.

Cluster 5 also contains the LL14B4_RS07875 and LL14B4_RS05330 (*phoH*) genes, which exhibit extremely increased activity (log_2_FoldChange > 3) and participate in signal transmission. The first gene encodes a receptor protein that responds to phosphate limitation in the environment [40], while the second is described as a Gram-positive signal peptide protein of the YSIRK family. The activation of these genes and their connection to *com* genes can be explained by the fact that signaling and quorum sensing activation play an essential role in the survival of bacterial populations under stress conditions, enabling a timely response and communication about environmental changes.

### 3.5. Regulatory Role of CpsY in Metabolic Reprogramming

Network analysis identified a putative transcription factor, CpsY, within Cluster 48 (Figure 4). This regulator exhibits extensive connectivity both within the cluster and with genes from other clusters. Under the *comX* overexpression condition, *cpsY* is repressed (log_2_FoldChange = −4.64), and the majority of genes associated with it also show decreased expression levels.

Previous studies have identified CpsY as a transcriptional regulator of multiple biological processes in various species. These include methionine metabolism, virulence in *Streptococcus iniae*, and a role in cell-wall stabilization through peptidoglycan acetylation [41]. In the reconstructed network, this TF shows a high number of interactions with *ilv* genes involved in branched-chain amino acid biosynthesis. These genes are known to interact with the CodY regulator within the same cluster (cluster 48), suggesting a potential indirect regulatory relationship between CpsY and CodY.

Furthermore, CpsY appears to link *ilv* genes with an uncharacterized cluster (Cluster 29), comprising the LL14B4_RS01800, LL14B4_RS01805, LL14B4_RS01810, LL14B4_RS01815, and LL14B4_RS01820 genes (Figure 4). While these genes are not assigned gene symbols in RefSeq, Pfam classification identifies them as *opp* genes. RefSeq functional descriptions indicate these genes encode transporter proteins belonging to a single operon. Additionally, this operon is enriched in three KEGG pathways: map01501 (beta-lactam resistance), map02010 (ABC transporters), and map02024 (quorum sensing). As *Lactococcus lactis* subsp. *lactis* is a Gram-positive bacterium, increasing cell wall permeability is essential for molecular uptake. Therefore, cell-wall remodeling and degradation systems may be required under conditions of competence activation.

Analysis of the common binding sites among the genes in Cluster 29, the regulator (*cpsY*), and the *ilv* genes revealed that the majority (>80%) of the genes possess CodY and CcpA binding sites. According to the literature, these two regulators repress the activity of *comX*. The presence of binding sites for these genes, along with the resulting changes in their activity, may point to a putative regulatory link during the competence state, though the exact causal relationship remains to be experimentally determined (Table S8).

### 3.6. Operon-Aware Reassessment of Key Functional Modules

Because bacterial genes are frequently transcribed as polycistronic operons, purely gene-level network inference can lead to edge inflation, where a single regulatory event at a shared promoter computationally manifests as multiple independent edges to each intra-operon gene. To ensure that our inferred regulatory modules reflect true regulatory architecture rather than redundant intra-operon co-expression, we performed a post hoc operon-aware reassessment. We mapped the GENIE3-derived targets of our primary findings—specifically, the WxL-associated cluster, the *opp* and *ilv* operons, the com genes, and the wider CpsY-associated module—onto the distinct operon structures predicted by Operon-mapper.

This analysis (Supplementary Table S10) revealed a highly biologically relevant topological pattern. For several key modules, including the opp and com operons, the putative regulators consistently exhibit high-confidence directed edges precisely targeting the primary lead genes (the main promoter regions) of these polycistronic units. Importantly, however, for other modules—such as the ilv operon and specific CpsY targets—the network captured strong regulatory edges targeting specific internal operon genes (e.g., the fifth or seventh gene in a seven-gene operon) rather than just the first gene. Rather than representing computational artifacts, such selective intra-operon targeting frequently reflects genuine, complex regulatory mechanisms, including the utilization of secondary internal promoters, partial transcript degradation, or condition-specific differential mRNA stability. Therefore, artificially collapsing the network into single operon nodes would have obscured these critical sub-operon regulatory dynamics. This reassessment confirms that the overarching regulatory relationships linking these transcription factors to their target functional operons remain structurally robust and that our gene-level topology accurately captures the nuanced biological reality of *L. lactis*.

## 4. Discussion

### 4.1. Performance and Reliability of the GENIE3-Inferred Network

The regulatory network of *Lactococcus lactis* was reconstructed using the GENIE3 method, which facilitated the identification of putative transcriptional regulators and their potential target genes. GENIE3 decomposes the network inference problem into multiple regression subproblems addressed through ensemble methods such as random forest. In the DREAM4 In Silico Multifactorial challenge simulated data, GENIE3 achieved the best results for bacterial gene network construction compared to other algorithms [19]. In addition, GENIE3 allows for the construction of networks using low numbers of input samples; as we found, only 38 appropriate samples in open sources, this aspect was decisive. Our network includes 70% of the *Lactococcus lactis* subsp. *lactis* genome, which depends on the number of samples, since GENIE3 detects only notably changing gene-expression activity, and to increase this number, it is essential to add more experimental variety. A persistent challenge in evaluating network inference for *Lactococcus lactis* is the absence of a comprehensive, experimentally validated “gold-standard” database of physical interactions (analogous to RegulonDB for *E. coli*), which precludes the calculation of an exact mathematical proportion of true-positive edges. Instead, the prediction accuracy of our network was assessed qualitatively through functional and biological benchmarking. First, the algorithm successfully recovered highly conserved, obligate co-regulatory units, serving as an internal positive control; for example, Cluster 18 assembled with near-perfect homogeneity for ribosomal proteins and translation machinery. Second, to directly address the need for biological validation without *de novo* experiments, we systematically compared our computationally inferred modules with experimentally characterized *Lactococcus* systems described by other authors in the published data. The algorithm successfully and blindly reconstructed several rigorously established regulatory circuits. For instance, our network accurately captured the established repressive effect of global regulator CodY on the *ilv* operon (BCAA biosynthesis) and the proteolytic *opp* system, perfectly aligning with prior experimental characterizations in *L. lactis* [11]. Furthermore, the network independently identified the CovRS two-component system as a master controller of the ComX competence regulon, corroborating recent in vivo mutational and transcriptomic validations by Toussaint et al. [10]. Additionally, the identification of conserved binding motifs for CcpA within our predicted metabolic modules matches the established carbon catabolite repression architecture characterized in wet-lab studies [12]. The successful recovery of these physically validated, literature-supported systems serves as a robust internal benchmarking metric. It provides strong confidence that the novel, uncharacterized nodes connected to these hubs (e.g., the WxL operon and CpsY) represent highly accurate and biologically meaningful predictions rather than computational artifacts.

It is important to note that the datasets integrated into our model originate from phenotypically diverse backgrounds, including *L. lactis* subsp. *lactis* and biovar *diacetylactis*. While integrating such heterogeneous strains inherently introduces genomic noise, unsupervised tree-based algorithms like GENIE3 utilize this exact variance to differentiate between core regulatory patterns conserved across all tested conditions and isolated experimental artifacts. The fact that the network successfully recovered highly conserved functional modules—such as the CodY regulon and the ribosomal clusters—across these varied strain backgrounds provides strong evidence that the identified regulatory hubs represent universal, core adaptive mechanisms rather than isolated phenotypic anomalies.

To facilitate systematic analysis, the Ward.D2 algorithm was applied to group genes into functional modules. The evidence that the selected clustering parameters of our gene network adequately reflect biological reality is the functionally united clusters. For instance, Cluster 18 consists almost exclusively of ribosomal genes. Additionally, there are other clusters that not only belong to a single operon or participate in a single metabolic process but also change their activity together, for example, the WxL cluster, the *opp* cluster, or the *pyr-car* clusters, including only genes of pyrimidine synthesis. The coordinated transcriptional responses observed within these clusters confirm that the inferred network architecture aligns with known physiological processes in *L. lactis*.

### 4.2. Multi-Layered Regulation of the Competence Master Regulator, ComX

Natural competence represents a significant adaptive mechanism that bacteria employ to respond to environmental stress via the uptake and integration of exogenous DNA. While the molecular basis of competence is well characterized in *Bacillus subtilis* and specific *Streptococcus* strains, comprehensive regulatory data for *Lactococcus lactis* remain limited.

In other Gram-positive bacteria, competence is known to alter the activity of hundreds of genes involved in diverse cellular processes, including amino acid and nucleotide metabolism and transport, cell-wall permeability, transport channels, energy metabolism, quorum sensing, and bacteriocin synthesis [38]. The stress signal is mediated by the competence-stimulating peptide (CSP), which is encoded by the *comC* gene [42]. The *comD* and *comE* genes encode a transmembrane histidine kinase and a response regulator, respectively, which transmit the signal into the cell and activate the *ComX* sigma factor. ComX functions as an integrative regulator linking external signals to competence development. According to studies in *Streptococcus pneumoniae*, ComX alters the activity of 240 genes [10].

The regulation of *comX* in *Lactococcus lactis* subsp. *lactis* involves an interconnected system of global transcription factors that function as repressors, including CodY, CcpA, and CovR [10]. Importantly, while our unsupervised computational approach independently identified the CovRS two-component system as a central hub linking environmental stress to the ComX-mediated competence regulon, this specific regulatory axis is strongly supported by recent experimental data [10] recently provided direct mechanistic evidence demonstrating that the CovRS system controls natural transformation in Lactococci. The ability of our unbiased GENIE3-based pipeline to successfully recover this newly established biological reality serves as a robust in silico validation of our methodology. Beyond transcriptional control, ComX levels are also modulated post-translationally through proteolytic degradation by the MecA-ClpCP complex. Because the co-expression network developed in this study is derived exclusively from transcriptomic data, it specifically captures these complex regulatory interactions at the transcriptional level, successfully reflecting this multi-layered regulatory architecture.

### 4.3. The CpsY-CodY Axis: Metabolic Reprogramming During Competence

Upon activation of the competence mechanism, the bacterial cell undergoes a systemic physiological transition directed toward resource conservation and survival under environmental stress. The reconstructed network reflects this transition through the significant repression of multiple biosynthetic pathways. Specifically, we observe downregulation of cluster 31 (*his* genes, histidine synthesis), cluster 38 (*met* genes, methionine synthesis), cluster 43 (*pyr*-*car* genes, pyrimidine synthesis), and cluster 48 (*ilv* genes, branched-chain amino acid synthesis). These observations indicate that competence induction modulates the regulators governing these primary metabolic processes.

One of the most interesting regulators is the potential transcription factor, CpsY, which is located in Cluster 48, together with *codY* and the *ilv* genes. The inferred interactions between CpsY and the *ilv* genes suggest a functional regulatory association. Furthermore, *cpsY* and its predicted targets exhibit the same activation pattern: their activity remains unchanged, except under the *comX* overexpression condition, where they are downregulated with a log_2_FoldChange below −4.8.

**Figure 7:**
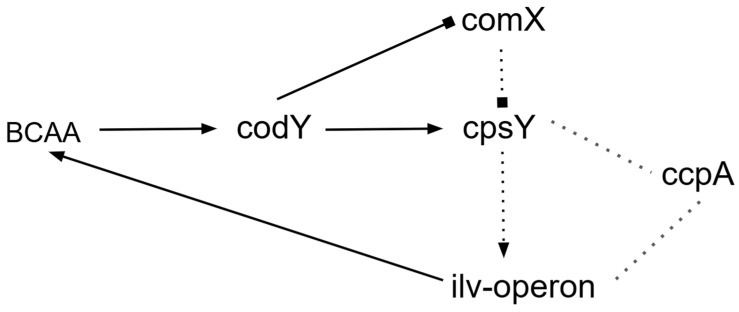
The potential mechanism of the loop inactivating codY during the competence state. Solid arrows indicate interactions described in the published data [10,43], and dashed arrows indicate potential interactions identified in this study. Arrows denote activating effects, while lines ending in squares represent repressive effects. Lines ending with open circles denote genes containing binding sites for transcription factors (with the circle oriented toward the target genes).

Notably, the *ilv* genes are responsible for the biosynthesis of branched-chain amino acids (leucine, isoleucine, and valine), and these amino acids act as triggers to activate CodY. Based on the matching activity patterns of CpsY and *ilv* genes, it can be suggested that CpsY regulates genes involved in branched-chain amino acid synthesis. Consequently, when *cpsY* expression decreases under conditions of *comX* overexpression, the expression of *ilv* genes also declines. This leads to a reduction in branched-chain amino acid synthesis, which, in turn, diminishes *codY* activity and relieves its repressive effect on *comX*. Furthermore, the analysis of shared binding sites revealed a common *ccpA* binding motif for the transcription factor (CpsY) and the *ilv* genes. We hypothesize that this regulatory circuit may function to sustain the competence state (Figure 7), though this predictive model requires subsequent mechanistic proof. Beyond amino acid biosynthesis, this global metabolic reprogramming is deeply intertwined with peptide scavenging. It is well established that CodY classically acts as a transcriptional repressor of the *L. lactis* proteolytic system—including the cell-envelope proteinase (*prtP*) and the *opp-pepO1* peptide transport operon—under nitrogen-rich conditions. Furthermore, the global regulator, CcpA, also plays a crucial role in modulating proteolysis and peptide transport, ensuring a tight coordination between carbon and nitrogen availability. As intracellular BCAA pools are likely to be depleted due to the repression of the *ilv* operon during competence, the subsequent relief of CodY-mediated repression would conventionally activate these proteolytic systems to scavenge exogenous resources. Indeed, our co-expression network reflects this broader proteolytic modulation, capturing various opp transporters (within Clusters 7 and 29) and peptidases (such as *pepO* and *pepX* linked to Cluster 11). The concurrent presence of CodY and CcpA binding motifs within these transporter modules (Table S8) underscores a highly integrated regulatory mechanism whereby the cell simultaneously coordinates competence, energy conservation, and peptide scavenging to survive environmental stress.

### 4.4. The WxL Operon: A Potential Key to Cell-Wall Permeability

Cell-wall permeability is a critical factor for the uptake of exogenous DNA. *Lactococcus lactis* subsp. *lactis*, a Gram-positive bacterium, possesses a dense peptidoglycan layer that acts as a physical barrier to DNA internalization. Quorum sensing mechanisms are concurrently induced during the competent state to facilitate signal perception—specifically, the detection of the competence-stimulating peptide (CSP).

In the reconstructed co-expression network, multiple clusters are enriched for COG categories corresponding to signal transduction mechanisms (T) and cell-wall biogenesis (M) or are described as membrane proteins. One of the most interesting findings is with respect to Cluster 11, which includes two completely undescribed genes and four genes encoding WxL domains according to the RefSeq annotation. It has already been shown in *Enterococcus faecalis* [20] that homologs of these WxL genes provide non-covalent binding of proteins directly to the peptidoglycan matrix.

Under *comX* overexpression, we observe that the entirety of Cluster 11 is downregulated, with an average log_2_FoldChange < −1.2. It is hypothesized that the transition to the competent state requires a temporary modification of the cell wall’s protein composition to facilitate DNA transport. Furthermore, the identification of a conserved binding motif within the WxL genes that resembles the PerR consensus sequence [32] suggests a regulatory link between oxidative stress responses and competence. The WxL operon may be controlled by a regulator participating in the coordination of both peroxide stress adaptation and the development of natural competence.

### 4.5. Resource Allocation: Transport Systems and Energy

Transcriptional alterations are observed in pathways associated with energy metabolism, reflecting the capacity of the cell to satisfy increased bioenergetic requirements under extreme environmental stress. Processes such as DNA recombination during the competent state necessitate substantial energy expenditure. In the reconstructed network, genes involved in energy metabolism exhibit significant upregulation. The *atp* operon, which is co-partitioned with the *com* genes, shows average log_2_FoldChange values exceeding 2.5. This operon encodes the ATP synthase complex responsible for the production of adenosine triphosphate, the primary chemical energy carrier.

Significant transcriptional changes were also identified in genes encoding transmembrane transport systems, including channels associated with xenobiotic efflux. Among these are the *opp* genes from Cluster 7. Rather than functioning in xenobiotic efflux, the *opp* operon encodes a specialized oligopeptide ABC transport system crucial for nitrogen uptake. Following transport, these imported peptides are degraded into usable amino acids by intracellular peptidases. As detailed in Section 4.3, this entire peptide-scavenging system is tightly regulated and classically repressed by CodY in nitrogen-rich environments. The network also captures uncharacterized ABC transporters in Cluster 29. Cluster 29 is topologically associated with *cpsY* and a putative transcription factor (LL14B4_RS08280), described in RefSeq as an XRE-family-like protein. It is important to clarify that while the acronym XRE (xenobiotic response element) historically originates from eukaryotic systems, bacterial XRE-family proteins constitute a massive, highly diverse class of transcriptional regulators. Rather than being restricted to xenobiotic efflux, these bacterial regulators are frequently co-opted to control core metabolic and developmental processes. For instance, data from *Pseudomonas putida* indicate that XRE-family proteins influence the D-branched-chain amino acid utilization pathway [44]. In *Streptomyces coelicolor*, another XRE-family gene affects both secondary metabolism and morphological development [45]. Based on these associations, we propose that putative transcription factor LL14B4_RS08280 contributes to the structural organization of the cell wall, given its connectivity to the WxL-domain genes in Cluster 11.

### 4.6. Comparative Insights and Hidden Nodes of the Network

When contextualizing our findings, it is essential to benchmark our co-expression network against existing regulatory models. A foundational regulatory framework for lactic acid bacteria was established by Ravcheev et al. [9] using comparative genomics and phylogenetic footprinting. While their pioneering approach excels at identifying highly conserved transcription-factor binding sites and regulon structures based on genomic sequences, it inherently reflects regulatory potential. In contrast, our study introduces a completely orthogonal, transcriptomically driven framework. By utilizing the GENIE3 random forest algorithm on a multi-stress RNA-seq compendium, our network captures the realized, condition-dependent regulatory activities that occur under specific environmental perturbations. Consequently, our approach complements the comparative genomics model: while sequence-based models map the physical wiring diagram, our co-expression network reveals the dynamic, physiological cross-talk between primary metabolism and stress adaptation (e.g., the CovRS and ComX axes). Ultimately, integrating both genomic predictions [9] and our functional transcriptomic network will be key to building highly accurate, dynamic metabolic models for *L. lactis*.

In summary, our results are consistent with published data, although we also identified novel potential genes that have not previously been described as components of the competence state in bacteria, along with their potential TFs. In addition, we confirmed the activation or repression of several processes in *Lactococcus lactis* subsp. *lactis* that have been described for our strains.

It is already known that energy metabolism decelerates during competence, as cellular growth and division are suspended [38]. In *Streptococcus suis*, competence induction occurs only in the early logarithmic growth phase and coincides with reduced expression of core metabolic pathways [46]. These activities resume once growth proceeds and competence ceases. A similar relation between competence and a transient state of growth arrest has been observed in *B. subtilis* and *S. mutans*, suggesting that competence development is functionally linked to the suspension of biosynthetic and cell-division processes in these species [47].

Our results show downregulation of genes responsible for the synthesis of histidine, leucine, isoleucine, valine, methionine, and pyrimidine. Notably, data from our co-expression network indicate that purine metabolism does not change under the *comX* overexpression condition. This contrast is significant, as other studies [48] have proposed that signals derived from purine metabolism modulate competence induction in *S. pneumoniae*. The stability of purine metabolism in *L. lactis* suggests that regulatory mechanisms may vary across different Gram-positive species.

The repressive effect of CodY on comX, mediated by branched-chain amino acids (BCAAs), is well documented. The reconstructed network identifies Cluster 48, which contains codY and the *ilv* operon. The latter is repressed and contains conserved CodY binding motifs. This indicates the established regulatory interaction between the CodY and ComX regulators within the context of the inferred network.

However, some of the identified genes and regulators have not been described in the context of the competence state in any bacterial strain. We detected upregulation of the ATP operon, which is responsible for ATP production, clarifying our understanding of energy metabolism during competence. Alongside the ATP operon, we discovered novel potential competence mechanisms in *Lactococcus lactis* subsp. *lactis*, including a WxL domain that may hypothetically modify the protein layer of the cell wall. The cell wall plays an important role—it must become permeable to allow molecules to enter. Furthermore, while biofilm formation and competence are often reported to have a negative correlation [43], the network identifies specific signaling components that may coordinate these transitions during the specific environmental stresses evaluated in our model (i.e., acid, thermal, and phage-induced stress).

Analyzing the WxL domain, we found that it is connected to LL14B4_RS03315, a TF belonging to the XRE protein family that has not been described as a player in competence. We propose that this regulator participates in the organizational control of cell-wall proteins and influences intercellular interactions. Another TF described previously but not in the context of competence is CpsY, which tightly interacts with Cluster 29, containing undescribed genes. These genes are enriched for ABC transporters and quorum-sensing processes. CpsY shares approximately 30% (e-value = 3 × 10^−42^) sequence identity with its homolog, CmbR, which was identified in *Streptococcus pneumoniae* [49]. While CmbR regulates cysteine and methionine metabolism in *Streptococcus* species, our results reveal that CpsY in *Lactococcus lactis* participates in the biosynthesis of branched-chain amino acids (BCAAs). Both Cluster 29 and the WxL operon contain CodY binding motifs, suggesting a high-level integration within the global regulatory hierarchy.

Taken together, these findings provide a computational guideline to identify new molecular targets for the genetic manipulation of *Lactococcus lactis* subsp. *lactis* and the development of strains with specific biotechnological properties.

### 4.7. Limitations and Future Directions

The predictive capacity and resolution of the reconstructed co-expression network are contingent upon the volume and environmental diversity of the underlying transcriptomic datasets. In the present study, the network was derived from a finite number of samples (38 diverse datasets), which precludes the use of traditional data-splitting cross-validation. In unsupervised network inference, artificially reducing the sample size severely compromises the biological variance required for robust random-forest tree building. Consequently, we utilized the entire aggregated compendium to maximize statistical power, relying on the inherent internal bootstrapping mechanism of the GENIE3 algorithm (1000 trees) to ensure edge robustness. While the network accurately captures regulatory interactions across the integrated conditions, its finite scope may limit the detection of links active only under unrepresented physiological states. Thus, the expansion of the data repository to include a broader range of experimental perturbations—such as oxidative stress, varying pH levels, or growth on alternative nutritional substrates—is essential to enhance network sensitivity and allow for future validation against completely independent transcriptomic compendia.

Furthermore, it is critical to acknowledge the inherent limitations of purely computational, gene-level network inference in bacteria. Methodologies relying on co-expression and topological edge ranking (e.g., GENIE3) do not establish direct physical interactions between transcription factors and their targets; an inferred edge may reflect direct DNA binding, indirect regulation, or shared downstream responses. Additionally, the polycistronic nature of bacterial transcription means that genes within the same operon exhibit high co-expression simply due to their shared transcriptional architecture. Treating these operon genes as independent targets can artificially inflate the number of regulatory edges. To mitigate this, we projected our inferred clusters onto computationally predicted operons. We observed that our highlighted regulatory hubs consistently target the primary promoter regions (lead genes) of these operonal units, suggesting that the broader functional modules remain robust despite intra-operon edge redundancy.

Ultimately, the interactions and transcription factors identified through this computational inference represent high-confidence functional hypotheses that require experimental verification. Future investigations should prioritize the biochemical and genetic validation of the proposed regulators, specifically the WxL operon and the CpsY-mediated regulatory circuit. The application of experimental techniques such as chromatin immunoprecipitation (ChIP-seq), electrophoretic mobility shift assays (EMSA), and targeted gene deletion studies is necessary to confirm the binding-site specificity and the functional roles of these components in vivo. Finally, the current network relies exclusively on transcriptomic data, which intrinsically limits overall model completeness. A purely RNA-based approach cannot capture post-transcriptional regulation, differential protein translation and degradation rates, or allosteric modulation by intracellular metabolites. For example, a global transcription factor such as CodY may be highly transcribed but functionally inactive without its specific metabolic ligands (e.g., branched-chain amino acids and GTP), leading to potential discrepancies between transcript co-expression and in vivo regulatory output. Because our model is currently blind to these metabolic feedback loops, our biological interpretations are strictly confined to the transcriptional layer of regulation. The future integration of diverse multi-omics data—incorporating quantitative proteomics to assess actual protein abundance and metabolomics to map allosteric feedback—will be vital to resolve these hidden regulatory layers and achieve a fully accurate, multi-scale systems model of *Lactococcus lactis* subsp. *lactis.*

## 5. Conclusions

This study represents a transcriptome-inferred, system-level reconstruction of the *Lactococcus lactis* subsp. *lactis* gene regulatory network, predicting putative regulatory modules for stress adaptation and natural competence. Based on our computational analysis, we proposed a novel cell wall-remodeling WxL operon (Cluster 11), uncovered the CpsY–CodY–Ilv metabolic axis (Cluster 48), and demonstrated the energetic coupling of DNA transformation under CovRS control (Cluster 5). While these regulatory hubs require direct experimental verification, they offer promising genetic targets for the engineering of robust industrial strains with specific biotechnological traits. For example, modulation of the newly identified CpsY–CodY–Ilv axis could optimize branched-chain amino acid metabolism, a pathway directly responsible for generating essential volatile flavor compounds in dairy fermentations. Similarly, targeted modifications of the cell-wall-associated WxL operon could alter surface properties to potentially improve phage resistance, while tuning the overarching CovRS-mediated stress network could enhance culture viability against industrial stressors such as rapid acidification or freeze-drying. Ultimately, our computational predictions provide a data-driven framework and a solid foundation of high-confidence hypotheses for follow-up in vivo validation and targeted metabolic engineering.

## Data Availability

The original data presented in the study are openly available in GitLab project at https://gitlab.sirius-web.org/students/2024/wolf.re/gene_network (accessed on 20 June 2026).

## Supplementary Materials

The following supporting information can be downloaded at: https://www.mdpi.com/article/10.3390/microorganisms14071594/s1, Table S1: Summary of predicted transcription factors in silico; Table S2: Description of the analyzed sequences; Table S3: The metadata for the genes included in the co-expression network; Table S4: log_2_FoldChange values for all conditions included in the work; Table S5: Description of the genes that interact with Cluster 11 (WXL); Table S6: Results of binding motif analysis in XSTREME for the WXL genes of Cluster 11; Table S7: Results of binding motif analysis in XSTREME for the 89 genes of Cluster 5, which includes com genes; Table S8: Results of binding motif analysis in XSTREME for the 10 genes: cpsY (TF), ilvABDN, and Cluster 29; Table S9: All software versions, parameters, and databases used in this study; Table S10: Post-hoc mapping of GENIE3 regulatory edges to Operon-mapper predictions for key functional modules (WxL, opp, com, ilv, and CpsY axes). Figure S1: Correlation between the samples used to construct the co-expression network; Figure S2: A scheme of *comX* regulation; Figure S3: A scheme of proteins involved in the late regulation of competence in *Lactococcus lactis*.

## Abbreviations

The following abbreviations are used in this manuscript:

ALE	Adaptive laboratory evolution
ATP	Adenosine triphosphate
BCAA	Branched-chain amino acid
ChIP-seq	Chromatin immunoprecipitation
COG	Clusters of Orthologous Groups
CSP	Competence-stimulating peptide
DEG	Differentially expressed genes
GO	Gene Ontology
GRAS	Generally recognized as safe
GRN	Gene regulatory network
KEGG	Kyoto Encyclopedia of Genes and Genomes
TF	Transcription factor
XRE	Xenobiotic response element

## References

[1] van Hylckama Vlieg J.E., Rademaker J.L., Bachmann H., Molenaar D., Kelly W.J., Siezen R.J. (2006). Natural Diversity and Adaptive Responses of *Lactococcus Lactis*. Curr. Opin. Biotechnol..

[2] Kazou M. (2022). Lactic Acid Bacteria: *Lactococcus Lactis*. Encyclopedia of Dairy Sciences.

[3] Zhang Z., Lv J., Pan L., Zhang Y. (2018). Roles and Applications of Probiotic *Lactobacillus* Strains. Appl. Microbiol. Biotechnol..

[4] Kleerebezemab M., Hols P., Hugenholtz J. (2000). Lactic Acid Bacteria as a Cell Factory: Rerouting of Carbon Metabolism in *Lactococcus Lactis* by Metabolic Engineering. Enzym. Microb. Technol..

[5] Song A.A., In L.L.A., Lim S.H.E., Rahim R.A. (2017). A Review on *Lactococcus Lactis*: From Food to Factory. Microb. Cell Fact..

[6] Zhang J., Fu R.-Y., Hugenholtz J., Li Y., Chen J. (2007). Glutathione Protects *Lactococcus Lactis* against Acid Stress. Appl. Environ. Microbiol..

[7] Panoff J.-M., Legrand S., Thammavongs B., Boutibonnes P. (1994). The Cold Shock Response in *Lactococcus Lactis* Subsp. *Lactis*. Curr. Microbiol..

[8] Whitaker R.D., Batt C.A. (1991). Characterization of the Heat Shock Response in *Lactococcus Lactis* Subsp. *Lactis*. Appl. Environ. Microbiol..

[9] Ravcheev D.A., Best A.A., Sernova N.V., Kazanov M.D., Novichkov P.S., Rodionov D.A. (2013). Genomic Reconstruction of Transcriptional Regulatory Networks in Lactic Acid Bacteria. BMC Genom..

[10] Toussaint F., de Frahan M.H., Poncelet F., Ladrière J.-M., Horvath P., Fremaux C., Hols P. (2024). Unveiling the Regulatory Network Controlling Natural Transformation in *Lactococci*. PLoS Genet..

[11] Guédon E., Sperandio B., Pons N., Ehrlich S.D., Renault P. (2005). Overall Control of Nitrogen Metabolism in *Lactococcus Lactis* by CodY, and Possible Models for CodY Regulation in Firmicutes. Microbiology.

[12] Gaudu P., Lamberet G., Poncet S., Gruss A. (2003). CcpA Regulation of Aerobic and Respiration Growth in *Lactococcus Lactis*. Mol. Microbiol..

[13] de Jong A., Hansen M.E., Kuipers O.P., Kilstrup M., Kok J. (2013). The Transcriptional and Gene Regulatory Network of Lactococcus Lactis MG1363 during Growth in Milk. PLoS ONE.

[14] Geng J., Wu J., Luo S., Liu D., Nie J., Fan G., Sun Q., Hu S., Wu L. (2025). Unveiling the Landscape of Prokaryotic Global Regulators through Deep Protein Language Models. mSystems.

[15] Zhang X., Zhao J., Hao J.-K., Zhao X.-M., Chen L. (2015). Conditional Mutual Inclusive Information Enables Accurate Quantification of Associations in Gene Regulatory Networks. Nucleic Acids Res..

[16] Terfve C., Cokelaer T., Henriques D., MacNamara A., Goncalves E., Morris M.K., van Iersel M., Lauffenburger D.A., Saez-Rodriguez J. (2012). CellNOptR: A Flexible Toolkit to Train Protein Signaling Networks to Data Using Multiple Logic Formalisms. BMC Syst. Biol..

[17] Garg A., Di Cara A., Xenarios I., Mendoza L., De Micheli G. (2008). Synchronous versus Asynchronous Modeling of Gene Regulatory Networks. Bioinformatics.

[18] Marbach D., Costello J.C., Küffner R., Vega N.M., Prill R.J., Camacho D.M., Allison K.R., Kellis M., Collins J.J., Stolovitzky G. (2012). Wisdom of Crowds for Robust Gene Network Inference. Nat. Methods.

[19] Huynh-Thu V.A., Irrthum A., Wehenkel L., Geurts P. (2010). Inferring Regulatory Networks from Expression Data Using Tree-Based Methods. PLoS ONE.

[20] Brinster S., Furlan S., Serror P. (2007). C-Terminal WxL Domain Mediates Cell Wall Binding in *Enterococcus Faecalis* and Other Gram-Positive Bacteria. J. Bacteriol..

[21] Tian K., Li Y., Wang B., Wu H., Caiyin Q., Zhang Z., Qiao J. (2019). The Genome and Transcriptome of *Lactococcus Lactis* Ssp. *Lactis* F44 and G423: Insights into Adaptation to the Acidic Environment. J. Dairy Sci..

[22] Dorau R., Chen J., Liu J., Ruhdal Jensen P., Solem C. (2021). Adaptive Laboratory Evolution as a Means to Generate *Lactococcus Lactis* Strains with Improved Thermotolerance and Ability to Autolyze. Appl. Environ. Microbiol..

[23] O’Connor P.B.F., Mahony J., Casey E., Baranov P.V., van Sinderen D., Yordanova M.M. (2024). Ribosome Profiling Reveals Downregulation of UMP Biosynthesis as the Major Early Response to Phage Infection. Microbiol. Spectr..

[24] Kim G.B., Gao Y., Palsson B.O., Lee S.Y. (2021). DeepTFactor: A Deep Learning-Based Tool for the Prediction of Transcription Factors. Proc. Natl. Acad. Sci. USA.

[25] Tenorio-Salgado S., Maya C.R., Galan-Vasquez E., Farias A.B., Álvarez-López D., Villalpando-Aguilar J.L., Martin A.J., Ledesma-Dominguez L., Perez-Rueda E. (2025). ENcyclopedia of TRAnscription Factors in Bacteria and Archaea Genomes (ENTRAF) Version 2.0. Database.

[26] Ortet P., De Luca G., Whitworth D.E., Barakat M. (2012). P2TF: A Comprehensive Resource for Analysis of Prokaryotic Transcription Factors. BMC Genom..

[27] de Sena Brandine G., Smith A.D. (2021). Falco: High-Speed FastQC Emulation for Quality Control of Sequencing Data. F1000Research.

[28] Chen S., Zhou Y., Chen Y., Gu J. (2018). Fastp: An Ultra-Fast All-in-One FASTQ Preprocessor. Bioinformatics.

[29] Langmead B., Salzberg S.L. (2012). Fast Gapped-Read Alignment with Bowtie 2. Nat. Methods.

[30] Love M.I., Huber W., Anders S. (2014). Moderated Estimation of Fold Change and Dispersion for RNA-Seq Data with DESeq2. Genome Biol..

[31] Sokolova T.S., Kolmykov S.K., Kulyashov M.A., Khlebodarova T.M., Kalyuzhnaya M.G., Akberdin I.R. (2025). Towards the Regulatory Network of Methanotrophs Based on Available Gene Expression Profiles. Microbiology.

[32] Shannon P., Markiel A., Ozier O., Baliga N.S., Wang J.T., Ramage D., Amin N., Schwikowski B., Ideker T. (2003). Cytoscape: A Software Environment for Integrated Models of Biomolecular Interaction Networks. Genome Res..

[33] Taboada B., Estrada K., Ciria R., Merino E. (2018). Operon-Mapper: A Web Server for Precise Operon Identification in Bacterial and Archaeal Genomes. Bioinformatics.

[34] Bailey T.L., Johnson J., Grant C.E., Noble W.S. (2015). The MEME Suite. Nucleic Acids Res..

[35] Siezen R., Boekhorst J., Muscariello L., Molenaar D., Renckens B., Kleerebezem M. (2006). *Lactobacillus Plantarum* Gene Clusters Encoding Putative Cell-Surface Protein Complexes for Carbohydrate Utilization Are Conserved in Specific Gram-Positive Bacteria. BMC Genom..

[36] Galloway-Peña J.R., Liang X., Singh K.V., Yadav P., Chang C., La Rosa S.L., Shelburne S., Ton-That H., Höök M., Murray B.E. (2015). The Identification and Functional Characterization of WxL Proteins from *Enterococcus Faecium* Reveal Surface Proteins Involved in Extracellular Matrix Interactions. J. Bacteriol..

[37] Fuangthong M., Herbig A.F., Bsat N., Helmann J.D. (2002). Regulation of the *Bacillus Subtilis* Fur and *perR* Genes by PerR: Not All Members of the PerR Regulon Are Peroxide Inducible. J. Bacteriol..

[38] Mulder J. (2021). Activation, Regulation and Physiology of Natural Competence in *Lactococcus Lactis*. Ph.D. Thesis.

[39] Delorme C., Godon J.J., Ehrlich S.D., Renault P. (1993). Gene Inactivation in *Lactococcus Lactis*: Histidine Biosynthesis. J. Bacteriol..

[40] Santos-Beneit F. (2015). The Pho Regulon: A Huge Regulatory Network in Bacteria. Front. Microbiol..

[41] Allen J.P., Neely M.N. (2012). CpsY Influences Streptococcus Iniae Cell Wall Adaptations Important for Neutrophil Intracellular Survival. Infect. Immun..

[42] Perry J.A., Jones M.B., Peterson S.N., Cvitkovitch D.G., Lévesque C.M. (2009). Peptide Alarmone Signalling Triggers an Auto-active Bacteriocin Necessary for Genetic Competence. Mol. Microbiol..

[43] Grandoni J.A., Zahler S.A., Calvo J.M. (1992). Transcriptional Regulation of the Ilv-Leu Operon of *Bacillus Subtilis*. J. Bacteriol..

[44] Fulton R.L., Downs D.M. (2025). The XRE Family Protein DbuR Is a Transcriptional Repressor of the Dbu Operon in *Pseudomonas Putida*. Appl. Environ. Microbiol..

[45] Zhu Y., Lu T., Zhang J., Zhang P., Tao M., Pang X. (2020). A Novel XRE Family Regulator That Controls Antibiotic Production and Development in *Streptomyces Coelicolor*. Appl. Microbiol. Biotechnol..

[46] Zaccaria E., van Baarlen P., de Greeff A., Morrison D.A., Smith H., Wells J.M. (2014). Control of Competence for DNA Transformation in *Streptococcus Suis* by Genetically Transferable Pherotypes. PLoS ONE.

[47] Shanker E., Federle M.J. (2017). Quorum Sensing Regulation of Competence and Bacteriocins in *Streptococcus Pneumoniae* and Mutans. Genes.

[48] Claverys J.-P., Havarstein L.S. (2002). Extracellular-peptide control of competence for genetic transformation in *Streptococcus pneumoniae*. Front. Biosci..

[49] Afzal M., Manzoor I., Kuipers O.P., Shafeeq S. (2016). Cysteine-Mediated Gene Expression and Characterization of the CmbR Regulon in *Streptococcus Pneumoniae*. Front. Microbiol..

