# Supplementary material for: Computational Identification of Novel Transcriptional Regulators and Functional Gene Clusters in Lactococcus lactis Using Integrated Bioinformatics Approaches
Source: Microorganisms. 2026 Jul 22;14(7):1594. doi: 10.3390/microorganisms14071594 (PMC13413889; doi:10.3390/microorganisms14071594)
Supplement: Supplementary file 1 [file microorganisms-14-01594-s001.zip › Supplementary_Figures.pdf]

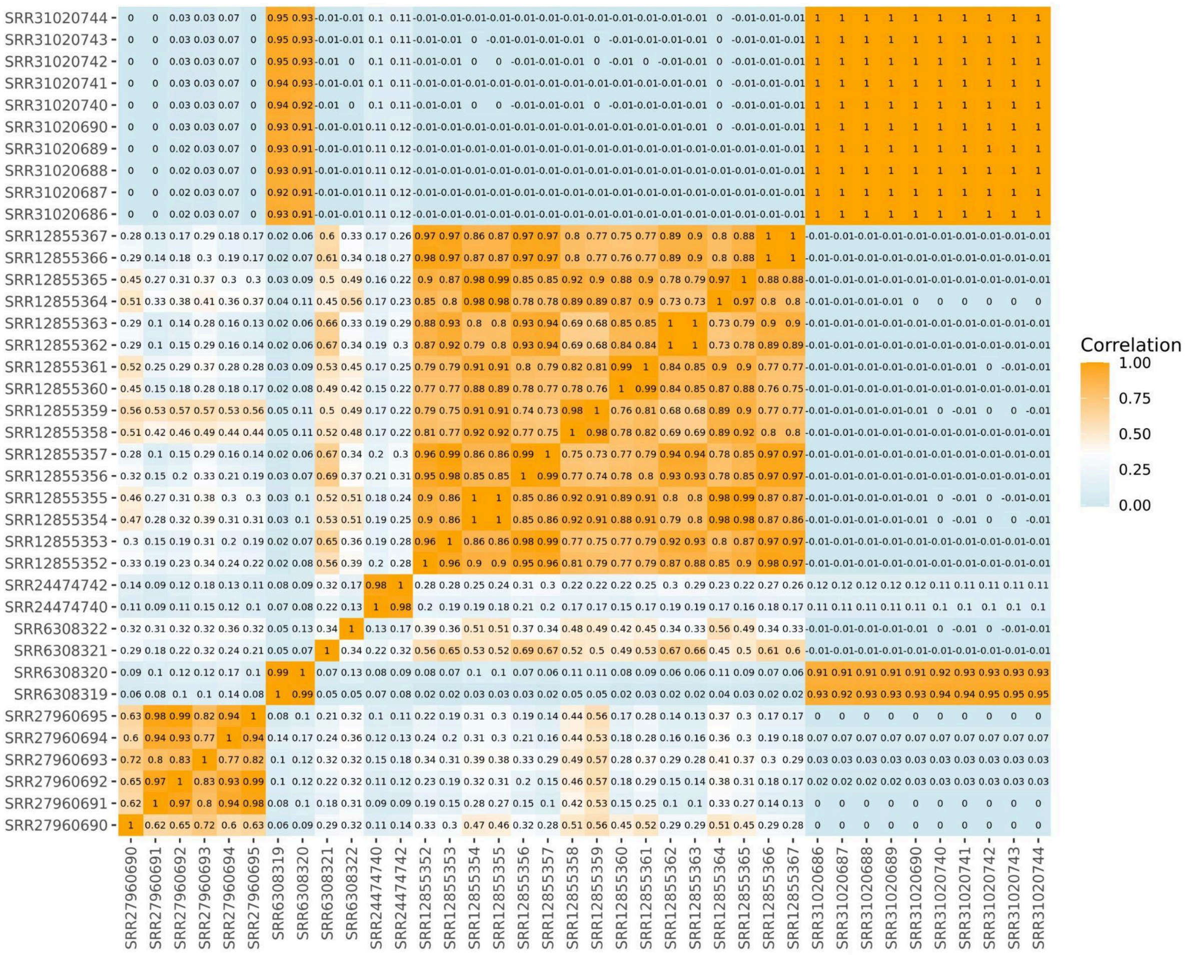

Supplementary Figure S1. Correlation between the samples used to construct the co-expression network.

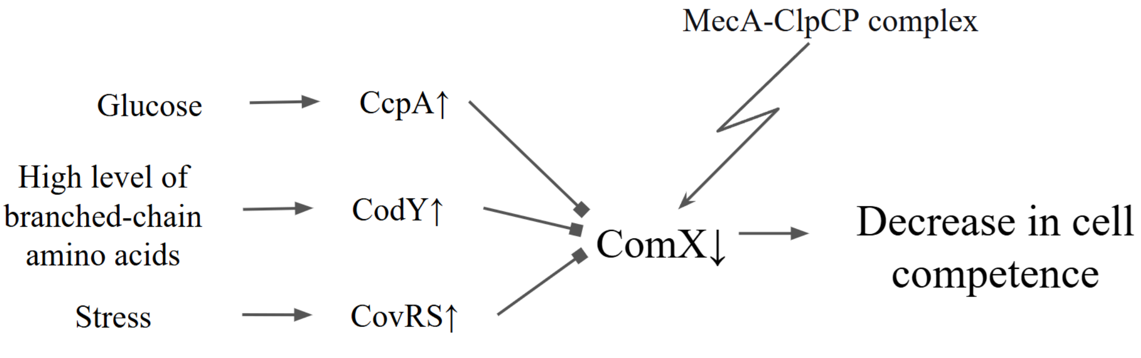

Supplementary Figure S2. A scheme of *comX* regulation. The regulators CcpA, CodY, and CovRS act as repressors of the *comX* factor, which activates

cellular competence. The concentration of these factors increases in the presence of glucose and high levels of branched-chain amino acids (BCAAs) (1,2).

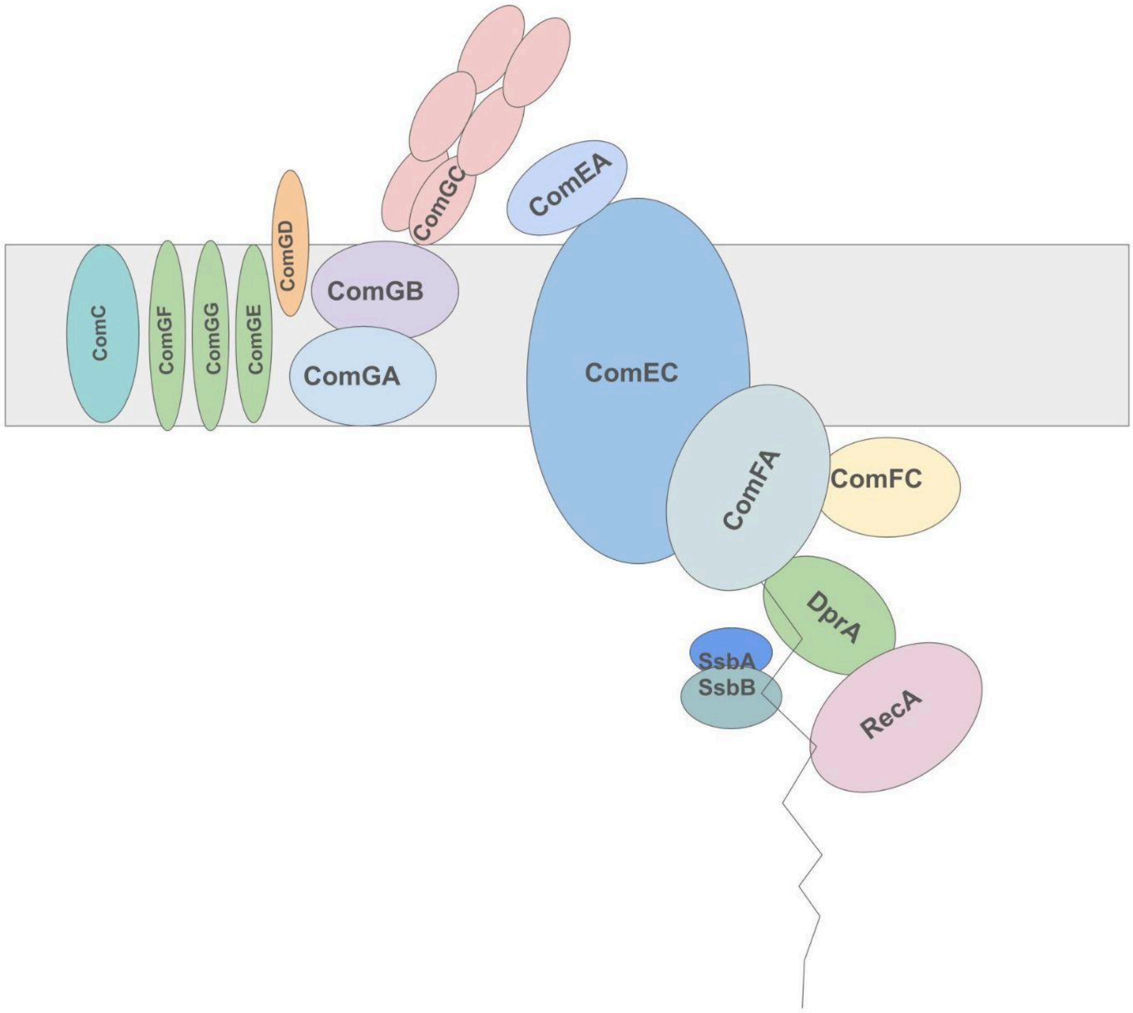

Supplementary Figure S3. A scheme of proteins involved in the late regulation of competence in *Lactococcus lactis*. ComG proteins decouple double-stranded DNA, after which the single-stranded DNA molecule enters the ComEA-ComEC-ComFA-ComFC complex, which transports it from the environment into the cell. The remaining proteins function to protect the DNA from degradation by cellular nucleases. Adapted from (3)

1. Toussaint F, Henry de Frahan M, Poncelet F, Ladrière JM, Horvath P, Fremaux C, et al. Unveiling the regulatory network controlling natural transformation in lactococci. PLoS Genet. 2024 Jul;20(7):e1011340. doi:10.1371/journal.pgen.1011340 PubMed PMID: 38950059; PubMed Central PMCID: PMC11244767.

2. Grandoni JA, Zahler SA, Calvo JM. Transcriptional regulation of the ilv-leu operon of Bacillus subtilis. J Bacteriol. 1992 May;174(10):3212–9. doi:10.1128/jb.174.10.3212-3219.1992

3. Mulder J. Activation, regulation and physiology of natural competence in Lactococcus lactis. [Groningen]: University of Groningen; 2021. doi:10.33612/diss.171825159
